# Reduced LOXL3 Expression Disrupts Microtubule Acetylation and Drives TP53-Dependent Cell Fate in Glioblastoma

**DOI:** 10.3390/cells15030219

**Published:** 2026-01-23

**Authors:** Talita de Sousa Laurentino, Roseli da Silva Soares, Antônio Marcondes Lerario, Ricardo Cesar Cintra, Suely Kazue Nagahashi Marie, Sueli Mieko Oba-Shinjo

**Affiliations:** 1 Cellular and Molecular Biology Laboratory (LIM 15), Department of Neurology, Faculdade de Medicina FMUSP, Universidade de Sao Paulo, Sao Paulo 01246-903, SP, Brazil; talitalaurentino@alumni.usp.br (T.d.S.L.); roseli.s@hc.fm.usp.br (R.d.S.S.); sknmarie@usp.br (S.K.N.M.); 2 Department of Internal Medicine, Division of Metabolism, Endocrinology and Diabetes, University of Michigan, Ann Arbor, MI 48109, USA; alerario@med.umich.edu; 3 Centro de Investigação Translacional em Oncologia, Instituto do Câncer do Estado de São Paulo (ICESP), Sao Paulo 01246-000, SP, Brazil; ricardocintra@alumni.usp.br; 4 Department of Radiology and Oncology, Faculdade de Medicina FMUSP, Universidade de Sao Paulo, Sao Paulo 05403-010, SP, Brazil

**Keywords:** glioblastoma, LOXL3, *TP53*, adhesion, apoptosis, senescence

## Abstract

Glioblastoma (GBM) is the most aggressive primary brain tumor, marked by molecular heterogeneity and poor clinical prognosis. Lysyl oxidase-like 3 (LOXL3) is frequently upregulated in GBM, but its mechanistic contribution remains insufficiently defined. Here, we investigated the functional role of LOXL3 in GBM using CRISPR-Cas9-mediated LOXL3 knockdown in two genetically distinct GBM cell lines: U87MG (wild-type *TP53*) and U251 (mutant *TP53*). Reduced LOXL3 expression markedly reduced α-tubulin acetylation, particularly in U87MG cells, and downregulated genes involved in cell cycle progression and proliferation. Both cell lines exhibited mitotic defects, including delayed cell cycle progression and spindle abnormalities; however, cell fate diverged according to *TP53* status. U87MG cells, sustained spindle checkpoint activation triggered a p53-dependent spindle checkpoint response culminating in apoptosis, while U251 cells underwent mitotic slippage and senescence. Transcriptomic analyses confirmed differential regulation of apoptosis versus senescence pathways in accordance with *TP53* functionality. Additionally, reduced LOXL3 expression markedly impaired adhesion and migration in U87MG cells, whereas U251 cells were minimally affected, consistent with more pronounced microtubule destabilization. Collectively, these findings identify that LOXL3 is a key regulator of microtubule homeostasis, mitotic fidelity, adhesion, and invasive behavior in GBM. Targeting LOXL3 may therefore provide a therapeutic opportunity for genotype-informed intervention in GBM.

## 1. Introduction

Glioblastoma (GBM) is the most common malignant primary tumor of the central nervous system (CNS) in adults, accounting for more than half of high-grade gliomas characterized by a median survival of only 12–15 months, despite multimodal therapy [1]. The intratumoral heterogeneity of GBM is driven by recurrent alterations in major oncogenic pathways, including p53, RB, and PI3K signaling, contributing to therapeutic resistance and limited clinical progress [2].

Modern molecular classification has refined these definitions, with the World Health Organization (WHO) 2021 criteria not strictly defining GBM as an isocitrate dehydrogenase (IDH)-wildtype entity [3]. Importantly, this diagnosis is increasingly driven by molecular signatures rather than histology alone. Diffuse astrocytic tumors that appear histologically lower-grade are now classified as GBM, CNS WHO grade 4 if they harbor *TERT* promoter mutations, *EGFR* amplification, or +7/−10 chromosome copy number changes [3]. *TP53* mutations are foundational to the IDH-mutant astrocytoma lineage, occurring in >90% of cases [4], while the frequency is only 25–30% of IDH-wildtype GBM [5], further underscoring the biological complexity that shapes GBM behavior and clinical outcomes.

Lysyl oxidase-like 3 (LOXL3), a copper-dependent amine oxidase of the lysyl oxidase family, is best known for catalyzing collagen and elastin cross-linking, contributing to extracellular matrix (ECM) stiffness and structural stabilization [6]. Beyond its canonical enzymatic function, LOXL3 participates in intracellular regulatory processes, such as STAT3 modulation through lysine deacetylation [7] and contributions to tumor development, progression, and therapy resistance across multiple malignancies [8,9,10]. In gliomas, LOXL3 expression increases with tumor grade and is higher in low-grade gliomas harboring wild-type IDH, a feature associated with poor prognosis [11]. In GBM, LOXL3 upregulation is associated with poor outcomes, showing a strong positive correlation with genes governing cytoskeletal organization, including tubulin-encoding transcripts, suggesting a functional relationship with microtubule (MT) dynamics [12].

MTs are the main structural component of the eukaryotic cytoskeleton and undergo continuous cycles of polymerization and depolymerization, a process termed dynamic instability. These processes are regulated by MT-associated proteins (MAPs) that bind to and stabilize the MT lattice [13]. These interactions are modulated by post-translational modifications of tubulin [14], with acetylation being particularly significant. Acetylation of α-tubulin, primarily catalyzed α-tubulin N-acetyltransferase 1 (α-TAT1), enhances MT stability by reducing breakage susceptibility, prolonging MT lifespan, and preventing structural damage [15,16]. Conversely, deacetylation is mediated by histone deacetylases HDAC5/6 and sirtuin 2 [17]. MT acetylation supports processes essential to cell survival, including mitotic spindle architecture, intracellular transport, and regulation of cell morphology, adhesion, and motility [13,16].

These observations raise the possibility that LOXL3 promotes GBM progression through the regulation of MT integrity. However, the mechanistic contribution of LOXL3 to cytoskeletal dynamics and cell fate in GBM remains largely undefined. Therefore, we investigated the functional role of LOXL3 in two genetically distinct GBM cell lines, U87MG (wild-type *TP53*) and U251 (mutant *TP53*), focusing on MT acetylation, cell cycle progression, ant tumor cell behavior. Our findings reveal that reduced LOXL3 expression disrupts MT homeostasis and proliferation, while *TP53* status determines whether cells undergo apoptosis or senescence. These results identify LOXL3 as a key regulator of GBM cell aggressiveness and support its potential as a molecular target for genotype-informed therapeutic strategies.

## 2. Materials and Methods

### 2.1. Cell Lines and Culture Conditions

Human GBM cell lines U87MG and U251 were obtained from American Type Culture Collection and authenticated by short tandem repeat (STR) profiling using GenePrint 10 System (Promega, Fitchburg, WI, USA). U87MG cells harbor wild-type *TP53*, whereas U251 cells carry *TP53* mutations [18]. This classification follows the WHO’s 2016 system [19]. Cells were cultured in DMEM supplemented with 100 U/mL penicillin and 100 μg/mL streptomycin (Thermo Fisher Scientific, Waltham, MA, USA), and 10% heat-inactivated fetal bovine serum (FBS; Cultilab, Campinas, SP, Brazil), at 37 °C in a 5% CO_2_. Mycoplasma contamination was routinely tested.

### 2.2. CRISPR-Cas9-Mediated LOXL3 Reduction

Two single-guide (sgRNAs) targeting exon 2 of *LOXL3* (NM_032603.4) were designed using an online platform [20]. sgRNA sequences targeting the coding exon 2 (Figure S1A) were cloned into the pSpCas9(BB)-2A-Puro V2.0 (PX459; Addgene, Cambridge, MA, USA) as described previously [21]. U87MG and U251 cells were transfected with 2 μg of plasmids (including empty vector [EV] controls), using FuGENE (Promega), and selected with puromycin (2.5 and 5 μg/mL, for U87MG and U251 cells, respectively, 72 h). Single-cell clones were isolated by limiting dilution.

### 2.3. DNA and RNA Extraction

Genomic DNA and total RNA were extracted using the AllPrep DNA/RNA Mini Kit (Qiagen, Valencia, CA, USA) according to the manufacturer’s instructions. Concentration and purity were determined by spectrophotometry. Samples with A260/A280 ratio greater than 1.8 were considered to have satisfactory purity.

### 2.4. Clone Validation

PCR amplification of the CRISPR-Cas9 *LOXL3* target region was performed with 100 ng of DNA in GoTaq Green Master Mix buffer containing 3 mM MgCl_2_, 1 U of GoTaq DNA polymerase (Promega), 10 µM primers, 2.5 µM dNTPs in a final volume of 25 µL. Amplicons were visualized on 2% agarose gel electrophoresis. Primer sequences are provided in Figure S1A. PCR products were subcloned into the pGEM-T Easy Vector (Promega), transformed into competent bacteria. PCR products were purified using Agencourt AMPure XP beads (Beckman Coulter Biosciences, Indianapolis, IN, USA) and sequenced using BigDye Terminator v3.1 Kit on an ABI 3500 Genetic Analyzer (Thermo Fisher Scientific). Sequences were aligned to the LOXL3 reference from GenBank.

### 2.5. Western Blotting

Cells were lysed in RIPA buffer (50 mM Tris-HCl 454, 1% NP-40, 0.25% Na-deoxycholate, 150 mM NaCl, 1 mM EDTA) supplemented with protease inhibitors (Sigma–Aldrich, St. Louis, MO, USA). Protein concentration was determined by the Bradford assay (Thermo Fisher Scientific). Equal amounts of protein (20 μg) were separated on 4–12% SDS-PAGE gels (NuPAGE, Thermo Fisher Scientific) and transferred to PVDF membranes (iBLOT, Thermo Fisher Scientific). Membranes were blocked with 5% non-fat milk and incubated overnight at 4 °C with primary Abs against LOXL3 (1:1000, rabbit; Aviva, San Diego, CA, USA), acetylated α-tubulin (1:1000, mouse; Sigma–Aldrich), α-tubulin (1:8000, mouse; Sigma–Aldrich), and β-actin (mouse, Sigma–Aldrich; 1:20,000). After washing, HRP-conjugated secondary Abs (1:1000; Sigma–Aldrich) were applied for 1 h at room temperature. Signal was detected using the Clarity Western ECL substrate (Bio-Rad, Hercules, CA, USA) and imaged on ImageQuant LAS4000 (GE Healthcare, Pittsburgh, PA, USA). Densitometry was performed using ImageJ v1.53 (National Institutes of Health, Bethesda, MD, USA). Two independent biological replicates were analyzed.

### 2.6. Immunofluorescence Microscopy

Cells grown on glass coverslips were fixed with 4% paraformaldehyde (15 min), permeabilized with 0.1% Triton X-100 (10 min), and blocked with 4% goat serum (30 min). Overnight incubation at 4 °C with anti-acetylated α-tubulin Ab (1:200; Sigma–Aldrich) was followed by Alexa Fluor 568-conjugated secondary Ab (1:400; Thermo Fisher Scientific). Nuclei were counterstained with DAPI (Thermo Fisher Scientific). Images were acquired by confocal microscopy (Zeiss 510 LSM META and 780-NLO; Carl Zeiss Microscopy, Thornwood, NY, USA) and analyzed with ImageJ v1.53.

### 2.7. RNA Sequencing and Bioinformatics Analysis

RNA-seq libraries were generated from quadruplicates of parental, control EV, and LOXL3-KD cells using QuantSeq 3′mRNA-Seq Library Prep Kit-FWD for Illumina (Lexogen, Vienna, Austria). Library size distribution was assessed with TapeStation 4200 (Agilent Technologies, Santa Clara, CA, USA). Pooled DNA libraries were sequenced on a NextSeq 500 (Illumina, San Diego, CA, USA) to a mean depth of 5 million single-end 75-bp reads per sample at SELA Facility Core, School of Medicine, University of Sao Paulo. Data (GEO accession number GSE288138) were processed using FASTQC for quality control [22], STAR for alignment to hg38 [23], and featureCounts for read quantification [24]. Counts per million (CPM) were normalized using edgeR v2 [25]. Differential expression was assessed using limma v3.50.3 following log_2_ CPM transformation [26]. Differentially expressed genes (DEGs; genes differentially expressed in LOXL3-KD cells relative to controls) (|log_2_FC| ≥ 0.5; adj. *p* ≤ 0.05) were analyzed using the Database for Annotation, Visualization and Integrated Discovery (DAVID, 2021 update) and the Gene Ontology enrichment (GO, biological process). Heatmaps were generated from z-score-transformed log_2_ CPM values. See Tables S1 and S2 for DEFs and enrichment data.

### 2.8. Cell Viability Assay

Cells (1 × 10^3^/well) were seeded in 96-well plates. Cell viability was assessed daily for 4 days using PrestoBlue Cell Viability Reagent (Thermo Fisher Scientific), with fluorescence recorded at 540/560 nm (GloMax-96 Microplate Reader, Promega). Background fluorescence from medium-only wells was subtracted. Each condition was tested in octuplicate in two independent experiments.

### 2.9. Cell Cycle Analysis

Cells (5 × 10^3^/well) were serum-starved overnight to synchronize cycling and re-stimulated with complete medium. At 12, 24, and 36 h, cells were fixed with 70% ethanol, washed, and stored at 4 °C. Samples were treated with RNase A (30 µg/mL; Sigma-Aldrich) and stained with propidium iodide (PI; 50 µg/mL). DNA content was analyzed by flow cytometry (FACS Canto II; BD Biosciences, San Jose, CA, USA). Cell cycle profiles were evaluated in triplicate from two independent experiments using FlowJo v10.

### 2.10. Nuclear Morphometric Analysis (NMA)

Cells were fixed and stained with DAPI, and nuclei were imaged using EVOS M5000 fluorescence microscopy (Thermo Fisher Scientific). Nuclear area, perimeter, and irregularity index were quantified using ImageJ v1.53. Nuclei were classified as normal, large, irregular, and small, with subdivisions into regular and irregular groups, as previously described [27]. Control EV (*n* = 64, *n* = 108) and LOXL3-KD (*n* = 198, *n* = 233) from U87MG and U251 cells were analyzed across two independent experiments.

### 2.11. Cell Adhesion Assay

Cells (5 × 10^4^/well) were seeded into 96-well plates and allowed to adhere for 3 h. Non-adherent cells were removed by three PBS washes. Attached cells were quantified using the PrestoBlue assay. Experiments were performed in octuplicate across two independent replicates.

### 2.12. Cell Migration Assay

Migration was evaluated using a wound-healing assay. Cells (8 × 10^4^/well) were seeded in 48-well plates and grown to 70–80% confluence. A scratch was made with a sterile pipette tip, debris was removed, and cells were cultured in DMEM with 1% FBS. Images were taken at 0, 6, 18, and 24 h using a phase-contrast microscope. Assays were performed in octuplicate across two independent experiments. Wound closures were quantified with ImageJ.

### 2.13. Apoptosis Assay

Cells (5 × 10^4^/well) were seeded in six-well plates (quadruplicate, two experiments) and treated with temozolomide (TMZ, 0.5 mM) or vehicle (DMSO). After 4 days, apoptosis was assessed using Annexin V-FITC/PI staining (Dead Cell Apoptosis Kit, Thermo Fisher Scientific) and analyzed by flow cytometry (30,000 events/sample; FACS Canto II; BD Biosciences). Populations were classified as viable, early apoptotic, late apoptotic, or necrotic, and data were analyzed using FlowJo v10.

### 2.14. Senescence Assay

Senescence-associated-β-galactosidase (SA-β-gal) activity was detected in cells (5 × 10^4^/well) cultured in six-well plates containing DMEM with 10% FBS. Cells were fixed with formaldehyde and glutaraldehyde (5 min, room temperature) and incubated with X-gal solution (1 mg/mL) overnight at 37 °C, as described previously [28]. Images were acquired by phase-contrast microscopy.

### 2.15. Statistical Analyses

Statistical analyses were executed using SPSS v20.0 (IBM Corporation, Armonk, NY, USA) and GraphPad Prism v8 (GraphPad Software, San Diego, CA, USA). Comparisons among multiple groups for tubulin expression, cell viability, cell cycle, cell adhesion, cell migration, apoptosis, and nuclear morphology were conducted using two-way analysis of variance (ANOVA) with Tukey’s post hoc test. Gene expression data were analyzed using one-way ANOVA with Tukey’s post hoc test or Student’s *t*-test. Z-score-normalized values were utilized to generate heatmaps. Data are presented as mean ± SD unless indicated otherwise. Statistical significance was set at *p* ≤ 0.05.

## 3. Results

### 3.1. CRISPR-Cas9-Mediated LOXL3 Reduction in Expression in GBM Cell Lines

**Figure 1:**
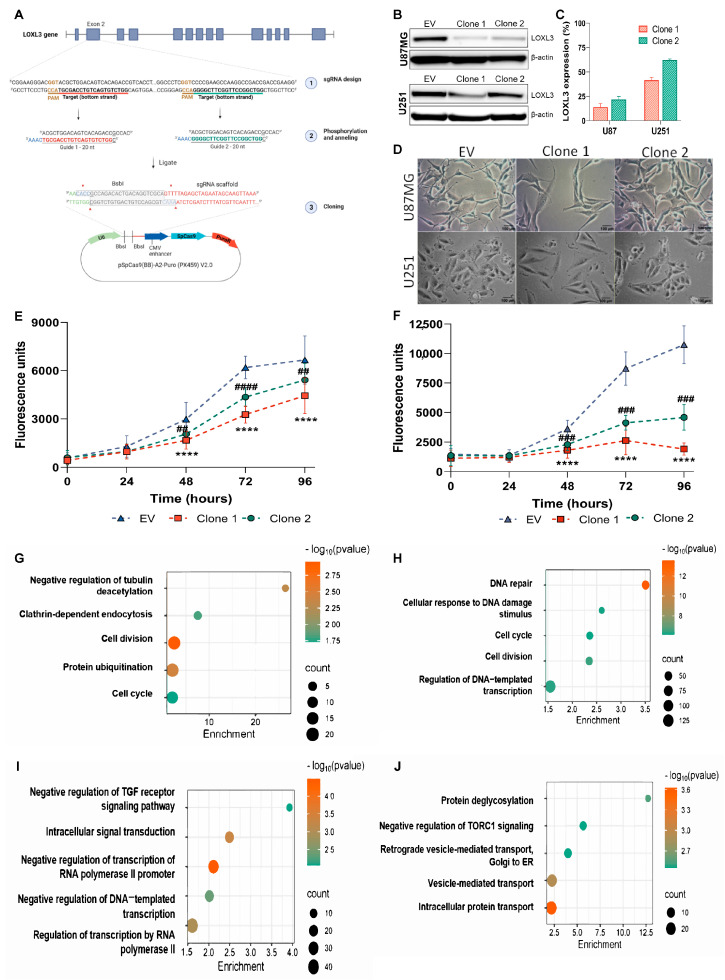
CRISPR-Cas9-mediated LOXL3 reduction in U87MG and U251 cell lines: (**A**) Schematic representation of the LOXL3 silencing strategy using CRISPR-Cas9 with two unique single guide RNAs (sgRNAs). (**B**) Western blot of LOXL3 protein levels in EV controls and LOXL3 partial knockout clones (clones 1 and 2). β-actin served as a protein loading control. LOXL3: 83 kDa; β-actin: 42 kDa. (**C**) Quantification of LOXL3 expression normalized to β-actin and EV controls in U87MG and U251 cells. Results are expressed as percentages relative to the EV controls (mean ± SD). (**D**) Representative phase-contrast images showing the morphological characteristics of EV controls and LOXL3-KD clones. (**E**,**F**) Cell viability of EV controls and LOXL3-KD clones in U87MG and U251 cells, respectively, measured over time. Data are presented as mean ± SD. Statistical significances of clones 1 and 2 versus EV controls at each time point: ## *p* ≤ 0.01, ### *p* ≤ 0.001, **** or #### *p* ≤ 0.0001. (**G**–**J**) RNA-seq analysis of LOXL3 partial knockout U87MG and U251 cells compared with EV controls. Top five enriched Gene Ontology biological processes for downregulated (**G**,**H**) and upregulated (**I**,**J**) genes in U87MG and U251 cells, respectively. EV, empty vector; SD, standard deviation.

CRISPR–Cas9 editing was employed to generate partial LOXL3-knockout (LOXL3-KD) clones in U87MG (wild-type *TP53*) and U251GBM (mutant *TP53*) cell lines to investigate the functional role of LOXL3 in GBM. Two sgRNAs targeting exon 2 of LOXL3 were cloned into the pSpCas9(BB) vector (Figure 1A) and transfected into cells. Following clonal selection, Sanger sequencing confirmed the introduction of heterozygous genomic alterations in all clones, including a nucleotide conversion (U87MG clone 1) and deletions of variable length (U87MG clone 2; U251 clones 1 and 2). Notably, none of the clones exhibited a complete knockout, and all retained one wild-type allele (Figure S1B).

Western blotting analysis identified clones exhibiting the most substantial LOXL3 protein reduction, designated as clones 1 and 2 for each cell line (Figure 1B). Densitometric quantification showed LOXL3 expression decreases to 13.9% and 21.8% of control in U87MG clones 1 and 2, and to 41.6% and 62.1% in U251 clones 1 and 2, respectively (Figure 1C). LOXL3-KD cells displayed visible enlarged morphology, which was most pronounced in clones with stronger LOXL3 suppression (Figure 1D).

LOXL3 reduced expression impaired cell proliferation in both cell lines, with clone 1 demonstrating the greatest effect. In U87MG clone 1 cell viability was reduced 1.7-, 1.8-, and 1.5-fold at 48, 72 and 96 h compared with control cells (Figure 1E). U251 clone 1 exhibited an even stronger phenotype with viability decreases of 2, 3.14, and 5.6-fold at the same time points (Figure 1F). Based on these results, subsequent analyses were conducted using clone 1 from each cell line, hereafter referred to as LOXL3-KD.

Transcriptomic profiling identified 921 DEGs in U87MG cells and 1974 DEGs in LOXL3-KD U251 cells (|log_2_FC| ≥ 0.5; adjusted *p* ≤ 0.05), including 520 and 1074 downregulated genes, and 401 and 900 upregulated genes, respectively (Table S1). GO enrichment indicated significant repression of pathways associated with cell cycle regulation (GO:0007049) and cell division (GO:0051301) in both cell lines (Figure 1G–J; Table S2). Additionally, LOXL3-KD U87MG cells showed reduced enrichment of negative regulation of the tubulin acetylation (GO:1904428), whereas LOXL3-KD U251 cells exhibited suppression of DNA repair (GO:0006281) and cellular response to DNA damage stimulus (GO:0006974). These data demonstrate successful LOXL3 suppression in both GBM cell lines and reveal a shared transcriptional signature linked to impaired proliferation and cell cycle progression, along with cell line-specific vulnerabilities associated with microtubule stability or DNA damage responses. Our results also revealed that LOXL3 is essential for DNA repair and cell division, positioning LOXL3 as a target for new therapies of IDH-wildtype GBM and IDH-mutant astrocytomas, WHO grade 4.

### 3.2. LOXL3 Partial Knockout Reduces α-Tubulin Acetylation

**Figure 2:**
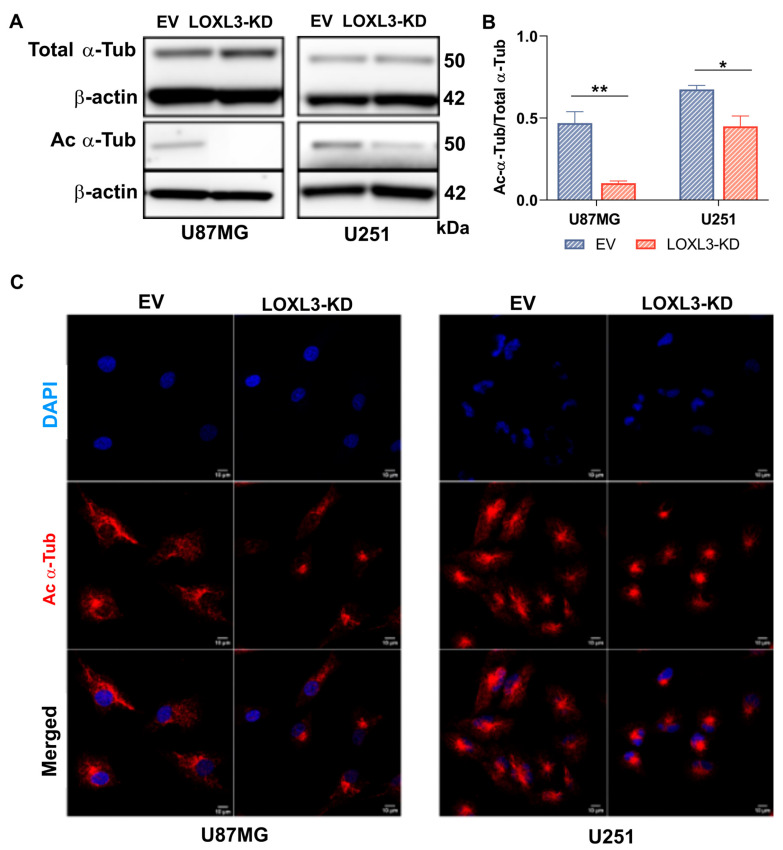
LOXL3 partial knockout reduces α-tubulin acetylation: (**A**) Representative Western blot of total (Total-α-Tub) and acetylated α-tubulin (Ac-α-Tub) in EV controls and LOXL3-KD. β-actin serves as a loading control. (**B**) Quantification of Ac-α-Tub relative to Total-α-Tub, normalized to β-actin and EV controls, of LOXL3-KD cells relative to EV controls. Data are mean ± SD. Statistical significances: * *p* ≤ 0.05, ** *p* ≤ 0.001. (**C**) Immunofluorescence images of nuclei (DAPI, blue) and Ac-α-Tub (red) in EV and LOXL3-KD U87MG and U251 cells. Images captured at 63× magnification with a 5× digital zoom. EV, empty vector; KD, partial knockout; and SD, standard deviation.

Based on transcriptomic enrichment linking LOXL3 to microtubule regulation, we next assessed α-tubulin acetylation. Western blotting revealed a marked reduction in acetylated α-tubulin levels in LOXL3-KD cells from both lines, with a substantially stronger decrease in U87MG cells (4.6-fold) than in U251 cells (1.5-fold) (Figure 2A,B). Immunofluorescence confirmed reduced acetylated microtubules in both models (Figure 2C). These results establish that LOXL3 positively regulates microtubule acetylation in GBM cells, establishing the LOXL3 axis as a requirement for microtubule stability and DNA damage response.

### 3.3. Reduced LOXL3 Expression Delays Cell Cycle Progression and Disrupts Mitosis

**Figure 3:**
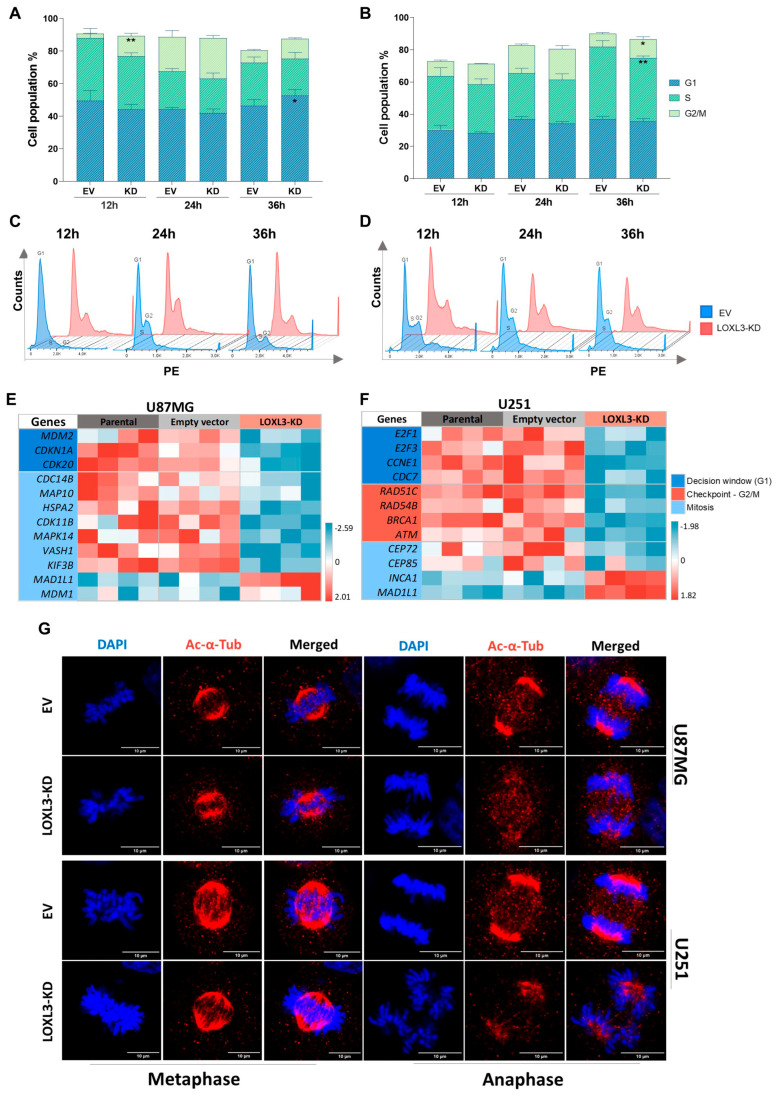
LOXL3 partial knockout disrupts cell cycle progression: (**A**,**B**) Percentage distribution of cells in G1, S, G2/M phases at 12, 24, and 36 h after synchronization in EV and LOXL3-KD U87MG and U251 cells. Analysis by flow cytometry and PI staining. Data are mean ± SD. Statistical significances: * *p* ≤ 0.05, ** *p* ≤ 0.01. (**C**,**D**) Representative histograms of cell cycle phase distribution. (**E**,**F**) Heatmaps of differentially expressed genes in parental, EV, and LOXL3-KD U87MG and U251 showing z-score of normalized log_2_ CPM. (**G**) Immunofluorescence of Ac α-Tub (red) and nuclei (DAPI, blue) in EV and LOXL3-KD cells during metaphase and anaphase, illustrating spindle organization. EV, empty vector; KD, partial knockout; PI, propidium iodide; CPM, counts per million; and SD, standard deviation.

Flow cytometry analysis following synchronization demonstrated that LOXL3 partial knockout impaired cell cycle progression in both cell lines but with distinct checkpoint responses. LOXL3-KD U87MG cells displayed transient G2/M accumulation at 12 h followed by G1 arrest at 36 h, whereas LOXL3-KD U251 cells predominantly accumulated in S and G2/M phases at 36 h (Figure 3A–D).

Consistently, transcriptomic profiling revealed a broad downregulation of cell-cycle-related genes in both cell lines (Figure 3E,F). In U87MG cells, downregulation of genes involved in p53 regulation (*MDM2*, *CDKN1A*, *MAPK14*), mitotic exit and G1 initiation (*HSPA2*, *CDC14B*), and microtubule-associated proteins (*MAP10*), together with spindle assembly checkpoint (SAC) activation (*MAD1L1* upregulation) and negative regulation of centriole duplication (MDM1 upregulation), suggested sustained mitotic surveillance. In contrast, U251 cells showed reduced expression of G1/S controllers (*E2F1*, *E2F3*, *CCNE1*, *CDC7*), DNA repair genes (*RAD51C*, *RAD54B*, *BRCA1*, *ATM*), and mitotic centrosome regulators (*CEP72*, *CEP85*), together with upregulation of *INCA1* (CDK inhibitor) and *MAD1L1*, implying checkpoint failure and genomic stress. Immunofluorescence imaging further confirmed mitotic spindle defects in both models, with U251 LOLX3-KD cells displaying pronounced spindle disorganization and increased multipolarity (Figure 3G). Together, these observations provide evidence of essential role of LOXL3 for mitotic fidelity and that its reduced expression triggers a *TP53*-dependent fate.

### 3.4. Reduced LOXL3 Expression Alters Nuclear Morphology and Differentially Modulates Cell Fate

**Figure 4:**
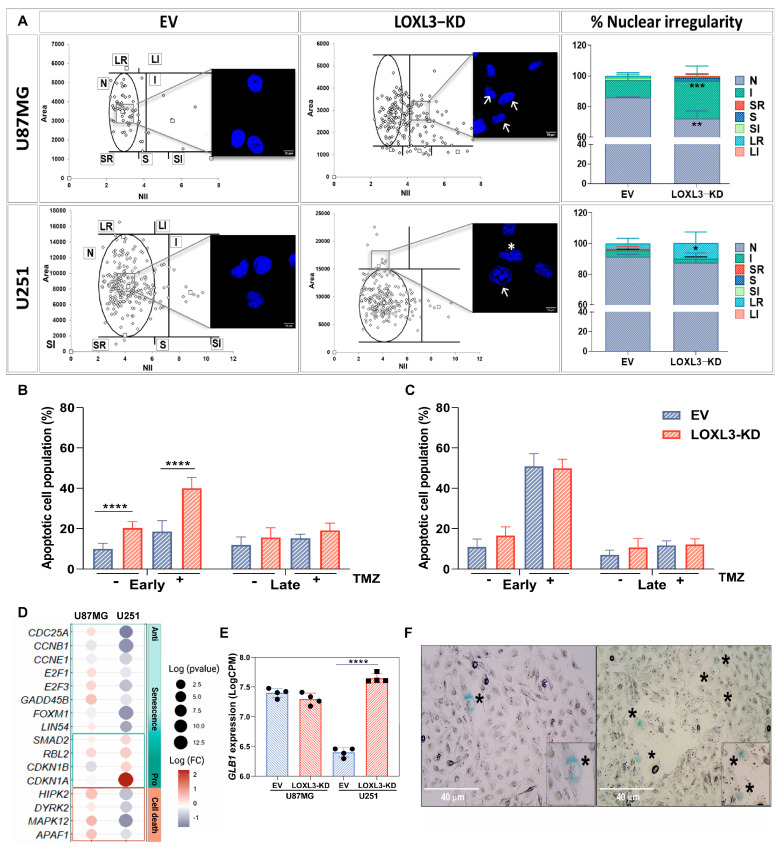
Nuclear morphometric analysis and cell death following LOXL3 reduction in expression. (**A**) Distribution of nuclear morphometric populations in EV controls and LOXL3-KD U87MG and U251 cells, based on nuclear irregularity index (NII). Each point represents a single nucleus. Bar graphs illustrate mean ± SD percentages of nuclear categories: normal (N), irregular (I), small and regular (SR), small (S), small and irregular (SI), large and regular (LR), and large and irregular (LI). Arrows indicate altered nuclei; asterisk denotes micronuclei. (**B**,**C**) Quantification of apoptotic cell populations in EV and LOXL3-KD U87MG and U251 cells, with or without TMZ treatment. Apoptosis assessed by annexin V/PI staining and flow cytometry. Bars indicate mean ± SD percentages of early and late apoptotic cells. (**D**) Dot plot of DEGs related to cell death and senescence in LOXL3-KD cells compared to EV controls. Dot size: −log_10_ (*p*-value); color: log_2_ fold change (FC). (**E**) Expression of GLB1 (β-galactosidase) in EV and LOXL3-KD U87MG and U251 cells. Statistical significances: * *p* ≤ 0.05, ** *p* ≤ 0.01, *** *p* ≤ 0.001, **** *p* ≤ 0.0001. (**F**) Representative images of senescence-associated β-galactosidase staining (*) in EV and LOXL3-KD U251 cells. EV, empty vector; KD, partial knockout; SD, standard deviation; TMZ, temozolomide; and DEG, differential expression of gene.

NMA identified distinct phenotypes in LOXL3-KD populations: U87MG cells showed irregular and fragmented nuclei, consistent with mitotic catastrophe, whereas U251 cells displayed enlarged, round nuclei, characteristic of senescence (Figure 4A). Apoptosis assays showed significantly increased early apoptotic cell fractions in LOXL3-KD U87MG cells under basal conditions, which were further enhanced by TMZ treatment (Figure 4B). By contrast, LOXL3-KD U251 cells did not display increased apoptosis, with or without TMZ treatment (Figure 4C).

Transcriptomic profiles supported these divergent fates. LOXL3-KD U87MG cells upregulated p53-dependent pro-apoptotic mediators (*HIPK2*, *DYRK2*, *MAPK12*, *APAF1*), whereas LOXL3-KD U251 cells downregulated anti-senescence drivers (*CDC25A*, *CCNB1*) and upregulated senescence-associated genes (*RBL2*, *CDKN1A*, *CDKN1B*) (Figure 4D). Correspondingly, GLB1 (β-galactosidase) expression was significantly elevated in LOXL3-KD U251 cells but unchanged in U87MG cells (Figure 4E). Moreover, SA-β-gal activity confirmed increased senescence in LOXL3-KD U251 cells (Figure 4F). These findings elucidate a bifurcation of terminal cellular fates, revealing LOXL3 attenuation triggers apoptosis in *TP53*-competent cells and irreversible senescence in *TP53*-deficient cells. This establishes LOXL3 inhibition as a universal therapeutic axis that bypasses the *TP53* mutational status.

### 3.5. LOXL3 Partial Knockout Impairs Cell Adhesion and Migration

**Figure 5:**
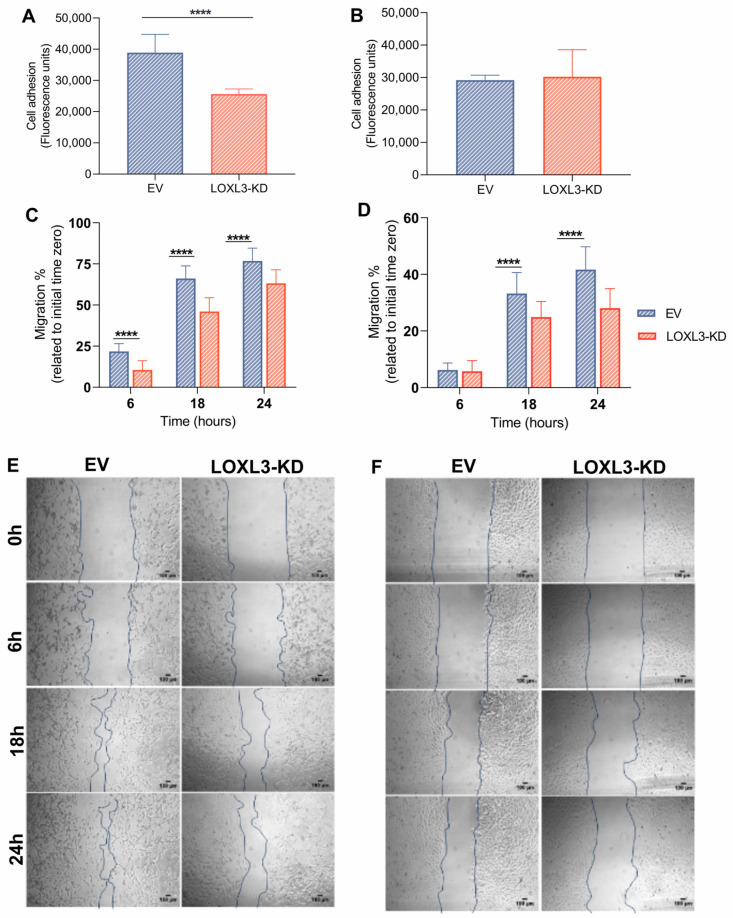
LOXL3 reduction impairs cell adhesion and migration: (**A**,**B**) Adhesion analysis of EV and LOXL3-KD U87MG and U251 cells. (**C**,**D**) Migration analysis by wound-healing assay at 6, 18, and 24 h post-scratch. Migration percentages relative to time zero for EV and LOXL3-KD U87MG (**C**) and U251 (**D**) cells. Bars indicate means ± SD. (**E**,**F**) Representative phase-contrast images (10× magnification) of wound-healing assay. Blue lines delineate scratch boundaries. Statistical significance: **** *p* ≤ 0.0001. EV, empty vector; KD, partial knockout; and SD, standard deviation.

Given the reduced microtubule acetylation in LOXL3-KD cells, we evaluated cytoskeleton-dependent behaviors. LOXL3-KD U87MG cells exhibited impaired adhesion (1.5-fold reduction; Figure 5A) and decreased migration across time (11.1%, 20.1%, and 13.6% reductions at 6, 18, and 24 h, Figure 5C,E). In contrast, U251 LOXL3-KD cells displayed no significant loss of adhesion (Figure 5B) and only modest impairments in migration at later points (8.3% and 13.6% at 18 and 24 h; Figure 5D,F). Analysis of cytoskeleton-related genes (GO:0005856) revealed 17 and 33 DEGs in LOXL3-KD U87MG and U251 cell lines, respectively. However, the ECM gene set (GO:0044420) yielded only minimal differential expression (one gene in U87MG; three genes in U251) (Figure S2). These findings suggest that LOXL3-regulated microtubule acetylation contributes to adhesion and motility, with a more substantial impact observed in U87MG cells, where acetylation loss is greater. Targeting LOXL3 opens a clear vulnerability to disrupt GBM’s invasive ability.

## 4. Discussion

### 4.1. LOXL3 as a Driver of GBM Aggressiveness

LOXL3 upregulation has been implicated in tumorigenesis and tumor progression, including viability, adhesion and invasion in GBM [11,29]. This study of CRISPR-Cas9-mediated knockout in two genetically distinct GBM cell lines, U87MG (wild-type *TP53*) and U251 (mutant *TP53*) cells, confirmed these associations, producing increased cell size, reduced viability, and impaired proliferation, consistent with our previous transient silencing study [12]. While *TP53* status profoundly influences cellular stress responses and cell fate decisions, it is not expected to directly affect the molecular efficiency of *LOXL3* knockdown. Differences in *TP53* status do not block the catalytic ability of Cas9 to induce DNA breaks; rather, *TP53* acts as a downstream modulator of the cellular phenotypic response to genomic stress rather than an upstream determinant of the molecular efficiency of CRISPR-Cas9-mediated gene editing [30]. Therefore, *TP53* status is likely to modulate the cellular interpretation of LOXL3 reduced expression—determining the severity of the growth arrest or apoptotic response—rather than the primary degree of gene silencing achieved [31].

Reductions in viability following LOXL3 silencing were also reported in melanoma cells, where knockdown via shRNA induced genomic instability, mitotic defects, and death by apoptosis [32]. Only heterozygous clones were obtained, all retaining one wild-type allele and partial reduction in LOXL3 protein expression, suggesting selective pressure against complete LOXL3 loss. This observation aligns with an *in vivo* study in mice, where homozygous deletion of Lolx3 resulted in perinatal lethality, highlighting the essential role of LOXL3 in cellular survival [33].

### 4.2. Microtubule Acetylation and Cell Cycle Control

Mechanistically, reduced LOXL3 expression impaired α-tubulin acetylation, destabilized MT, and activated SAC. Tubulin acetylation is known to enhance MT flexibility, protect against structural damage, and support polymerization [13,34]. Transcriptomic analyses revealed downregulation of MT-stabilizing genes (*MAP10*, *HSPA2*, *CDC14B*) [35,36,37] in U87MG cells, suggesting that impaired MT acetylation disrupts G2/M arrest and subsequent G1 arrest. U251 cells showed altered G1/S regulators (*E2F1*, *E2F3*, *CCNE1*, *CDC7*) [38,39] and reduced homologous recombination DNA repair gene expression (*RAD51C*, *RAD54B*, *BRCA1*, *ATM*) [40,41,42,43]. These changes indicate compromised genomic stability and disrupted cell cycle progression, underscoring the role of LOXL3 in maintaining MT integrity and mitotic fidelity.

### 4.3. SAC Activation and Spindle Defects

Both LOXL3-KD cell lines exhibited SAC activation, a surveillance mechanism that delays mitosis until all chromosomes are correctly attached to MTs [44]. MT disruption is known to activate and prolong SAC, resulting in mitotic arrest [45]. In both cell lines, *MAD1L1*, encoding the SAC component MAD1, was upregulated. In addition, LOXL3-KD U87MG cells upregulated *MDM1*, an MT-binding protein that negatively regulates centriole duplication [46]. On the other hand, U251 cells displayed multipolar mitosis, consistent with excessive SAC activity and p53-independent mitotic slippage [47]. These findings highlight the importance of LOXL3 in spindle organization and checkpoint regulation.

### 4.4. Divergent Cell Fate Outcomes

GBM cell fate outcome diverged according to TP53 status. In LOXL3-KD U87MG cells, prolonged mitotic arrest triggered mitotic catastrophe and apoptosis via caspase activation [48], enhanced by TMZ, with transcriptomic evidence of p53-dependent pro-apoptotic signaling (*HIPK2*, *DYRK2*, *APAF1*) [49,50], alongside downregulating CDKN1A and MDM2 [51]. In contrast, LOXL3-KD U251 cells underwent senescence [52], characterized by flattened morphology, β-galactosidase activity [53], and upregulation of *CDKN1A*/*CDKN1B* (p21/p27), consistent with p53-independent CDK inhibition [54,55].

### 4.5. Impact on Adhesion and Migration

Partial LOXL3 knockout impaired adhesion and migration, with a stronger effect in U87MG cells, consistent with their higher knockout efficiency and greater loss of MT acetylation. These observations align with our previous study [12]. Given the role of MT acetylation in focal adhesion and motility of astrocytes [56], and LOXL3 expression correlation with *CTNNB1* (β-catenin) and SNAIL pathway activation in low-grade astrocytomas [11] and hepatocellular carcinoma [8], these results suggest that LOXL3 supports GBM invasiveness through MT-dependent cytoskeletal remodeling. Whether the canonical role of LOXL3 ECM crosslinking contributes to the observed phenotypes remains an open question. Nonetheless, our data suggest that the primary impact of LOXL3 in GBM is centered on the maintenance of intracellular structure integrity. While the expression of ECM genes remained unchanged across both cell lines, the internal cellular architecture was profoundly compromised following LOXL3 reduction. This cytoskeleton destabilization was characterized by a decrease in α-tubulin acetylation and the concomitant downregulation of key cytoskeletal regulatory genes in both U87MG and U251 cells. Consequently, LOXL3 appears to play a non-redundant role in stabilizing the GBM cytoskeleton. This function is distinct from that of other lysyl oxidase family members, particularly LOX, which is described as the predominant driver of ECM stiffening in various malignancies [57] and exhibits the strongest correlation with the ECM genes in GBM [11].

### 4.6. Therapeutic Implications

**Figure 6:**
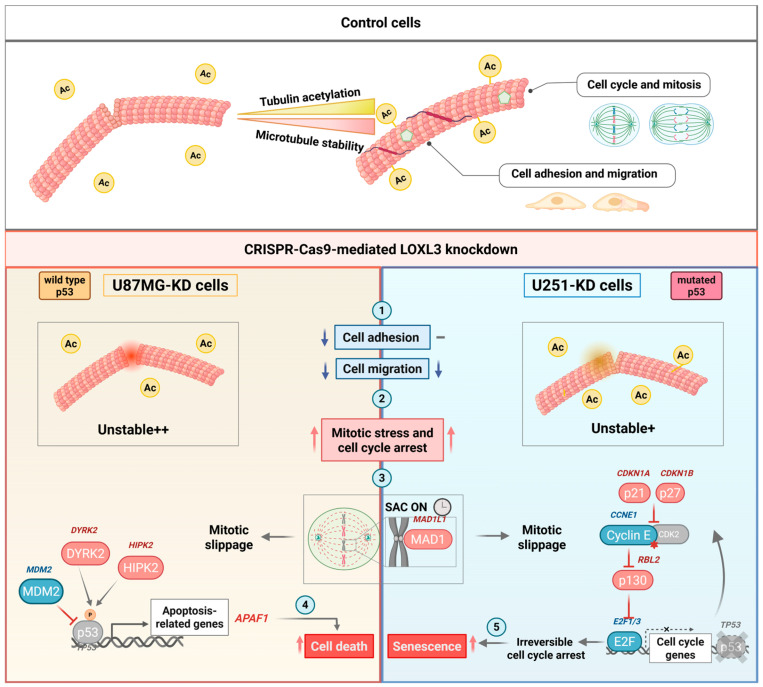
Summary of reduced LOXL3 expression effects in GBM cell lines. Tubulin acetylation, a post-translational modification, enhances microtubule stability and supports key cellular functions such as mitosis, adhesion, and migration. LOXL3 knockdown (KD) reduces tubulin acetylation in both U87MG and U251 cell lines, more strongly in U87MG cells. Functional consequences of lower LOXL3 expression included the following: ① reduced cell adhesion (U87MG only) and migration (both lines); ② mitotic stress and cell cycle arrest (both cells); ③ spindle assembly checkpoint (SAC) activation with increased MAD1 expression (*MAD1L1*) in both cell lines. SAC ensures accurate chromosome segregation and delays cell cycle progression. Extended SAC activation results in mitotic slippage, potentially leading to p53-dependent apoptosis in U87MG cells or irreversible cell cycle arrest and senescence in p53-mutated U251 cells; ④ in U87MG cells (wild-type *TP53*), delayed cell cycle progression, upregulation of p53-dependent apoptotic genes (*HIPK2*, *DYRK2*, *APAF1*) and downregulation of MDM2; ⑤ in U251 cells (mutant *TP53*), increased expression of senescence markers *CDKN1A* (p21) and *CDKN1B* (p27), downregulation of CCNE1, upregulation of *RBL2* (p130), and reduced *E2F1*/3 expression. Expressions of gene and protein changes are color-coded: red for upregulation, blue for downregulation. Arrows indicate functional increases (red) or decreases (blue) observed in cell assays. Created in BioRender. Laurentino, T. (2026) https://BioRender.com/9m5otw3.

Collectively, our findings establish LOXL3 as a critical regulator of MT acetylation, mitotic fidelity, and cell fate in GBM. Reduced LOXL3 expression destabilizes MT, activates SAC, and drives TP53-dependent outcomes—apoptosis in wild-type TP53 cells and senescence in *TP53*-mutant cells—while reducing invasiveness. This phenotype parallels the effects of benzimidazole carbamate compounds and other MT-targeting drugs, which induce G2/M arrest and mitotic catastrophe in GBM cells, leading to variable fates such as mitotic death, slippage, or senescence [58]. Therefore, the balance between mitotic catastrophe/apoptosis and senescence is dictated by p53 functionality, as summarized in Figure 6. Given that *TP53* mutations occur in approximately 25–30% of GBM, IDH-wildtype [5] and up to 90% of astrocytoma, IDH-mutant, grade 4 cases [4], LOXL3 inhibition may be therapeutically exploited in both genetic contexts. Future studies should validate these findings in orthotopic and patient-derived models, clarify enzymatic versus non-enzymatic contributions, and explore synergy with TMZ, microtubule-targeting drugs that inhibit acetylation or senolytic agents.

## 5. Conclusions

LOXL3 emerges as a multifunctional contributor to GBM aggressiveness, integrating structural and regulatory roles in MT dynamics, mitotic surveillance, and invasive capacity. By converging on pathways of MT destabilization and mitotic catastrophe, LOXL3 inhibition offers a mechanistic bridge to existing MT-targeting strategies, while its TP53-dependent divergence in cellular response underscores the importance of genomic context in therapeutic exploitation, supporting LOXL3 as a promising molecular target for genotype-informed combinational strategies.

## Data Availability

The original data of RNAseq presented in this study will be available in Gene Expression Omnibus (GEO), at https://www.ncbi.nlm.nih.gov/geo/query/acc.cgi, accession number GSE288138.

## Supplementary Materials

The following supporting information can be downloaded at https://www.mdpi.com/article/10.3390/cells15030219/s1, Figure S1: CRISPR–Cas9-mediated LOXL3 knockout validation. (A) Sequences of single-guide RNAs (sgRNAs) and primers used for LOXL3 knockout via CRISPR-Cas9. (B) Characterization of LOXL3 mutations generated by CRISPR-Cas9 in clones 1 and 2 of U87MG and U251 cells; Figure S2: Heatmaps of differentially expressed genes of cytoskeleton and extracellular matrix in parental, EV, and LOXL3-KD U87MG (A) and U251 (B) cells showing z-score of normalized log2 CPM; Table S1: Differentially expressed genes in LOXL3 partial knockout cells. List of upregulated and downregulated genes in LOXL3 partial knockout clones compared to empty vector (EV) controls in U87MG and U251 cells. Data include gene symbols, functional annotations, log_2_ fold change (FC), *p*-values, and adjusted *p*-values; Table S2: Gene ontology enrichment analysis. Top five enriched biological processes identified by gene ontology (GO) analysis for upregulated and downregulated genes in LOXL3 partial knockout U87MG and U251 cells. Pathways are ranked by *p*-value and include representative genes contributing to each term.

## Abbreviations

The following abbreviations are used in this manuscript:

GBM	Glioblastoma
LOXL3	Lysyl oxidase-like 3
MT	Microtubule
LD	Linear dichroism
SAC	Spindle assembly checkpoint
KD	Partial knockout
CRISPR–Cas9	Clustered Regularly Interspaced Short Palindromic Repeats–CRISPR-associated protein 9
*TP53*	Tumor protein p53 gene
EV	Empty vector
DEG	Differentially expressed gene
GO	Gene ontology
RNA-seq	RNA sequencing
NMA	Nuclear morphometric analysis
