# Supplementary figures and images for: Reduced LOXL3 Expression Disrupts Microtubule Acetylation and Drives TP53-Dependent Cell Fate in Glioblastoma
Source: Cells. 2026 Jan 23;15(3):219. doi: 10.3390/cells15030219 (PMC12896791; doi:10.3390/cells15030219)

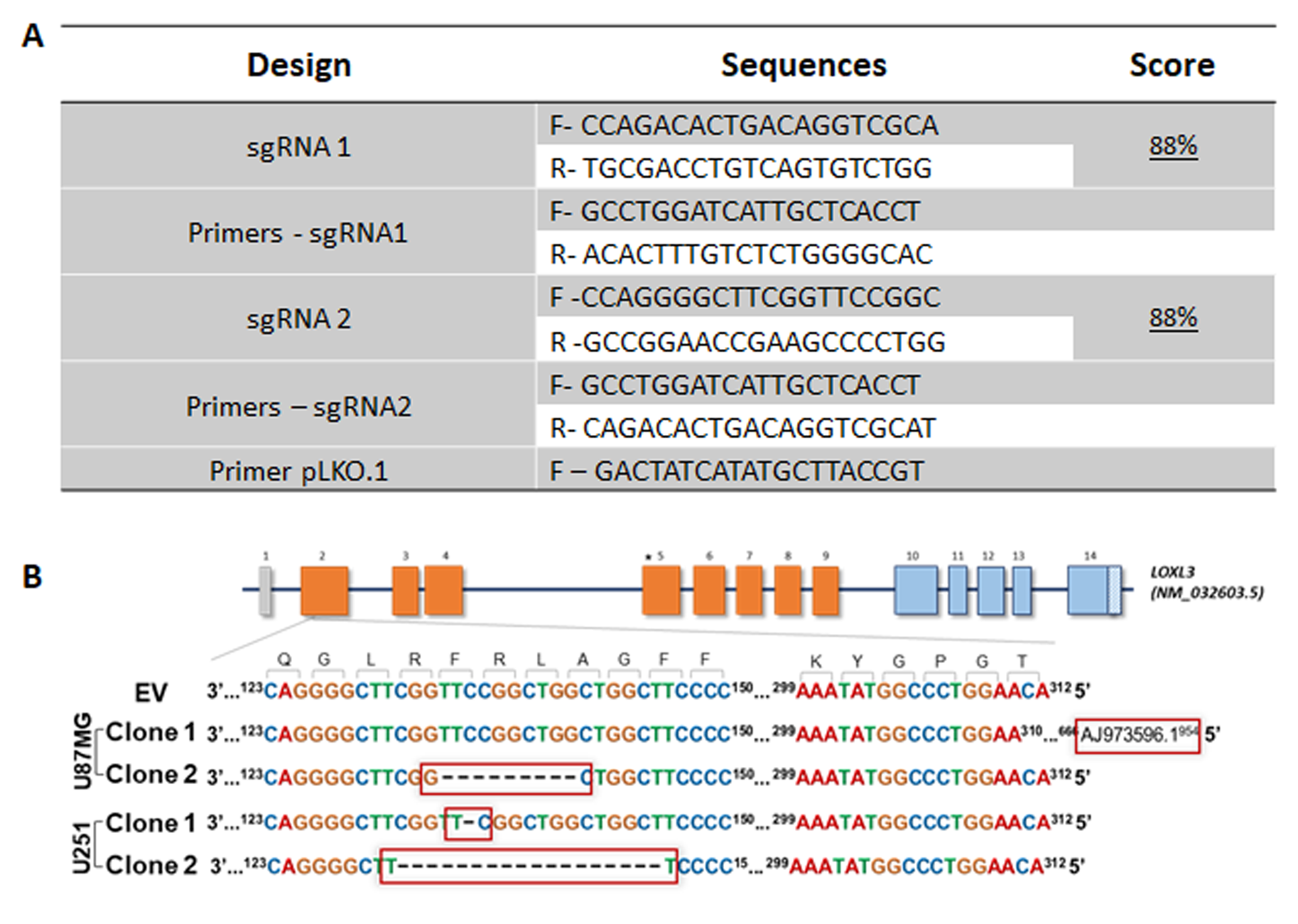

Supplement: Supplementary file 1 [file cells-15-00219-s001.zip › Figure S1.tif]

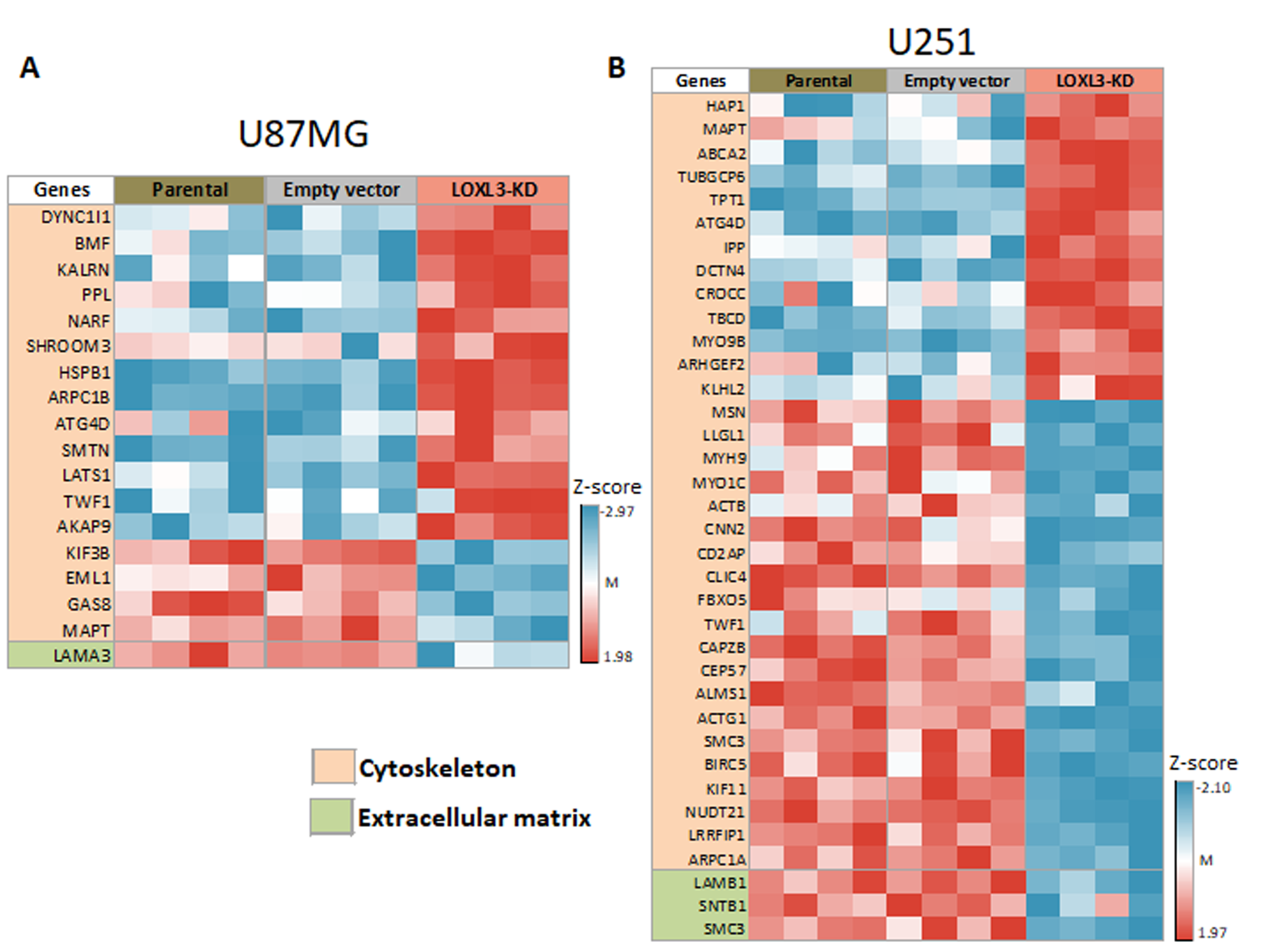

Supplement: Supplementary file 1 [file cells-15-00219-s001.zip › Figure S2.tif]
